# Pregnancy‐Associated Maternal Mortality Within One Year After Childbirth: Population‐Based Cohort Study

**DOI:** 10.1111/1471-0528.17985

**Published:** 2024-10-23

**Authors:** Nadia Arshad, Rolv Skjærven, Kari Klungsøyr, Linn Marie Sørbye, Liv Grimstvedt Kvalvik, Nils‐Halvdan Morken

**Affiliations:** ^1^ Department of Global Public Health and Primary Care, Faculty of Medicine University of Bergen Bergen Norway; ^2^ Department of Clinical Neuroscience Karolinska Institute Stockholm Sweden; ^3^ Centre for Fertility and Health Norwegian Institute of Public Health Oslo Norway; ^4^ Division for Mental and Physical Health Norwegian Institute of Public Health Bergen Norway; ^5^ Faculty of Health and Social Sciences Western Norway University of Applied Sciences Bergen Norway; ^6^ Norwegian Research Centre for Women's Health Oslo University Hospital, Rikshospitalet Oslo Norway; ^7^ Department of Obstetrics and Gynaecology Haukeland University Hospital Bergen Norway; ^8^ Department of Clinical Science University of Bergen Bergen Norway

**Keywords:** last pregnancy, maternal mortality, population‐based cohort, pregnancy complications

## Abstract

**Objective:**

The objective of this study is to assess associations between pregnancy complications and pregnancy‐associated maternal mortality (PAM) within 1 year after childbirth.

**Design:**

Population‐based cohort study.

**Setting:**

Norway, 1967–2020.

**Population:**

1 237 254 mothers with one or more singleton pregnancies registered in the Medical Birth Registry, 1967–2019 and followed in the Cause of Death Registry to 2020.

**Method**s**:**

Logistic regression was used to calculate odds ratios (ORs) with 95% confidence intervals (CIs), adjusted for maternal education, age, year of first childbirth and chronic medical conditions.

**Main Outcome Measures:**

PAM by lifetime history of pregnancy complications: placental abruption, preeclampsia, preterm birth, perinatal death, small for gestational age (< 2.5 percentile), gestational diabetes and gestational hypertension.

**Results:**

Crude OR for PAM was 4.24 (95% CI 3.53–5.10), if complications occurred in the last pregnancy, whereas 2.52 (2.08–3.06) if complications occurred in the first pregnancy, compared to mothers without complications in any pregnancy. Adjusted ORs for PAM when complications occurred in the last pregnancy were, for placental abruption 3.75 (1.20–11.72), preeclampsia: 4.42 (3.17–6.15), preterm birth: 4.32 (3.25–5.75), perinatal death: 24.18 (16.66–35.08), small for gestational age: 2.90 (1.85–4.54), gestational diabetes: 1.43 (0.63–3.25) and pregnancy hypertension: 2.05 (1.12–3.74) compared to mothers without complications. The OR for PAM increased slightly by increasing the number of complicated pregnancies but the trend was stronger for increasing number of complications in the last pregnancy (e.g., during 1999–2019: one complication; 4.14 [2.79–6.13], two complications; 11.50 [6.81–19.43]).

**Conclusion:**

Complications in the last pregnancy were more strongly associated with PAM than those in the first pregnancy.

## 1 Introduction

In most high‐income countries, obstetric care has markedly improved in recent decades, leading to a substantial decline in maternal mortality due, at least in part to institutional births, accessible antenatal care and effective identification and management of high‐risk pregnancies [1, 2, 3]. The maternal mortality ratio (MMR), defined as the number of maternal deaths occurring within 42 days after delivery per 100 000 livebirths [4], fell from 5 to 2 deaths per 100 000 from 2003 to 2017 in Norway [5].

The World Health Organization (WHO) defines maternal mortality as ‘annual number of female deaths from any cause related to or aggravated by pregnancy or its management (excluding accidental or incidental causes) during pregnancy and childbirth or within 42 days of termination of pregnancy, irrespective of the duration and site of the pregnancy’ [6]. The Centers for Disease Control and Prevention and the American Congress of Obstetricians and Gynaecologists (ACOG) coined the term ‘pregnancy‐associated mortality (PAM)’, including deaths during pregnancy or within 1 year of the end of pregnancy irrespective of the cause [7]. This study uses the ACOG definition for maternal mortality.

A previous study on preeclampsia and later‐life maternal cardiovascular mortality demonstrated that the association was modified by the number of lifetime pregnancies [8]. To the best of our knowledge, no study has examined how complications occurring across all of a woman's pregnancies may be associated with the risk of maternal death within 1 year after childbirth. In a linked pregnancy and mortality dataset, with the mother as the unit of observation, we aimed to estimate the association between lifetime history of pregnancy complications and maternal death within 1 year.

## 2 Methods

### 2.1 Data Source

Data were obtained from the population‐based Medical Birth Registry of Norway (MBRN). Since 1967, the MBRN has used mandatory notification to collect data on all live‐and stillbirths and pregnancy losses from 16 weeks of gestation, including information on maternal characteristics, medical and reproductive history before and during pregnancy, complications during pregnancy and delivery and pregnancy outcomes. During prenatal visits, at delivery and until hospital discharge, data are registered by the attending midwife and obstetrician using a standardised notification form, either as free text before 1999 or by check boxes in addition to free text from 1999 [9]. A Swedish study showed that introduction of check boxes for collecting registry data has improved sensitivity [10]. The revised MBRN notification form, used from 1999, included ultrasound‐based gestational age and maternal smoking [11]. Medical conditions, including pregnancy complications, were diagnosed by physicians, and free text was coded using the International Classification of Disease (ICD) (version 8: 1967–1998 and version 10: 1999–2020) [12]. Several MBRN variables have been validated [13], for example, preeclampsia had a positive predictive value of 83.9% [14].

The National Population Register in Norway provides a unique identification number to all residents in the country. The MBRN is routinely matched with the Population Register and receives all identification numbers, dates of birth and death. The identification number enabled linkage of all pregnancies to their mothers. The proportion of livebirths captured by the MBRN is nearly 100% [15]. Data from the MBRN were linked to data from the Norwegian Cause of Death Registry for maternal deaths and to the National Education Database at Statistics Norway for information on the mothers' highest attained level of education.

### 2.2 Study Population

The study population was restricted to mothers with a registered first singleton birth (liveborn or stillborn) in the MBRN during 1967–2019 and followed for death as registered in the Cause of Death Registry to June 2020. Mothers with multiple births (*n* = 39 669) were excluded as these pregnancies are associated with complications that may differ with regards to underlying aetiology.

We used different subject criteria in our analyses. For the main analysis, we included women whose first birth occurred 1967–2019. However, when evaluating PAM by periods (1967–1982, 1983–1998 and 1999–2019), we restricted the study population to women whose first childbirth occurred prior to 2013, so that all study subjects had at least 7 years for additional childbirth [8]. We divided the year of last childbirth into three periods to capture changes in reporting format and obstetric practices over time. These cutoffs were chosen because ultrasound examination became part of clinical practice in Norway in the early 1980s and a revised birth notification form was introduced in the MBRN in 1999.

### 2.3 Exposure

Lifetime history of pregnancy complications included placental abruption, preeclampsia (preterm and term), preterm birth, perinatal death, small for gestational age (SGA), gestational diabetes mellitus (GDM) and gestational hypertension. Placental abruption was indicated by a check box or as a diagnostic code according to the ICD version 8 (632.1 and 651.4), and ICD‐10 (O45) [12]. Preeclampsia was registered using the clinical definitions being used at the year of birth. The definition has been based on clinical diagnosis reflecting the criteria corresponding to the Norwegian Society of Gynaecology and Obstetrics [16]. The consistent criteria across the study period have been new‐onset hypertension and proteinuria. Cases registered with eclampsia and HELLP (haemolysis, elevated liver enzymes and low platelet count) syndrome were categorised as preeclampsia in our study. Cases with registered hypertension during pregnancy were considered g*estational hypertension* [17]. Preterm birth was defined as delivery < 37 completed weeks of gestation and, *perinatal death* included stillbirth (≥ 16 weeks) or early neonatal death within 7 days after delivery [9]. In a sensitivity analysis, we used the WHO standard definition of perinatal death [18] including stillbirth (≥ 22 weeks) or early neonatal deaths within 7 days after delivery. *Gestational age* was based on the first day of the last menstrual period (LMP) and reported in completed weeks. From 1999, gestational age was based on ultrasound estimation if LMP was missing or if LMP‐based gestational age differed from ultrasound estimates by > 10 days. For pregnancies conceived by in vitro fertilisation, gestational age was based on the date of embryo transfer plus 14 days [19]. *SGA* was defined as sex‐specific birthweight by gestational age below the 2.5th percentile according to the Norwegian standard [20]. Though we acknowledge that SGA cutoffs may be arbitrary [21], we chose the 2.5th percentile aiming to exclude constitutionally small babies. *GDM* was indicated by a check box or as diagnostic codes according to the ICD‐8 (250) and ICD‐10 (O244) versions from the early 1980s. GDM was clinically diagnosed, according to the National guidelines by the Norwegian Society of Gynaecology and Obstetrics [22].

### 2.4 Outcome

The outcome was maternal death within 1 year after childbirth, corresponding to the ACOG definition of PAM.

### 2.5 Covariates

Maternal age at first birth (categorised as ≤ 20, 21–24, 25–29, 30–34, 35–39 and ≥ 40 years), highest attained education at last childbirth (primary [< 11 years], higher‐secondary [11–13 years], and university‐college [≥ 14 years]) [23], period of last childbirth (1967–1982; 1983–1998; 1999–2019) and maternal chronic medical conditions (asthma, rheumatoid arthritis, epilepsy, renal disease, pregestational hypertension and diabetes) were considered as potential confounders.

Missing data were < 2% for maternal education, and 2.3% for maternal country of birth, among mothers who were alive 1 year after their last childbirth. Among mothers who died, 19.4% had missing information on country of birth and 9.6% on education (Table S1). Mothers with missing education were excluded in models adjusting for education. Several sensitivity analyses were performed.

### 2.6 Statistical Analyses

We used Logistic regression to estimate odds ratios (ORs) with 95% confidence intervals (CIs) for associations between pregnancy complications and risk of maternal death within 1 year. The OR is a good approximation of the risk ratio (relative risk) when the outcome is rare [24]. Maternal mortality was estimated for each pregnancy complication if occurring in the first, last and in any pregnancy, compared to mothers with no complications in any pregnancy (reference). We also estimated risk of maternal death in models stratified by parity and complications. For some women, more than one complication occurred in the same pregnancy. We examined the risk of maternal death by occurrence of one, two, or three complications in the last pregnancy. For analyses by periods of list childbirth (1967–1982, 1983–1998 and 1999–2019), reference categories were (a) mothers without complications in each period (1967–1982; 1983–1998 and 1999–2019), (b) mothers without complications (1999–2019) as a common reference and (c) same reference as in ‘b’, obtaining adjusted estimates for the last period only. Analyses were conducted in Stata version 18.5.

The study was approved by the Regional Committee for Medical and Health Research Ethics (REK VEST 2020/13818) on 25.10.2021 with an exemption from written informed consent. Patients were not involved in the development of the research and core outcome sets were not used. The funder of the study had no role in the study design, data collection, analyses, interpretation or write‐up of the results.

## 3 Results

**FIGURE 1:**
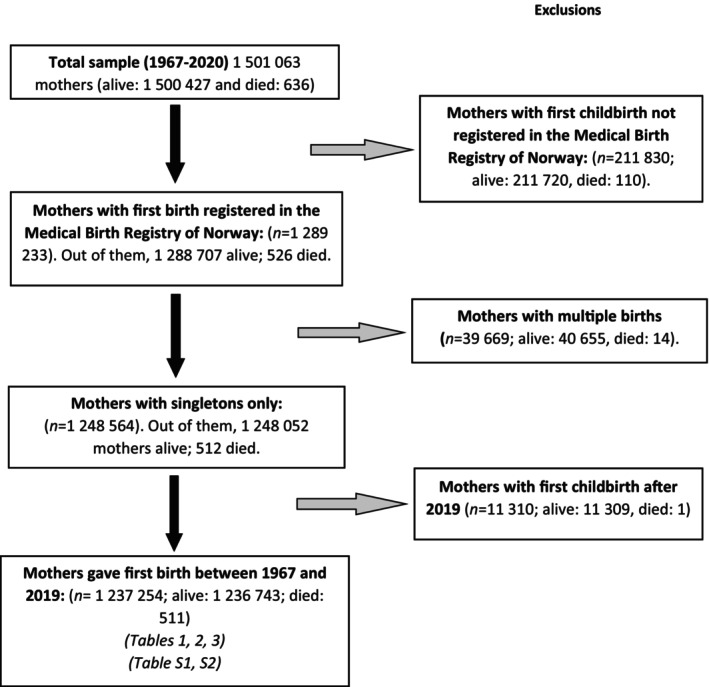
Flow chart of the study population.

Of 1 501 063 mothers who gave birth between 1967 and 2020, we excluded 211 830 mothers whose first childbirth was not registered in the MBRN, as for these women we could not examine their complete childbirth history, resulting in 1 289 233 mothers, of whom 526 died within first year after delivery. Our final cohort included 1 237 254 mothers with first singleton births in the MBRN between 1967 and 2019. Among them, 511 died within the first year after childbirth (Figure 1).

Mortality within 1 year after childbirth for mothers with first singleton births in Norway between 1967 and 2013, decreased across time periods. Relative to the last period (1999–2019), the ORs were 1.64 (95% CI 1.31–2.05) for the first (1967–1982) and 1.41 (95% CI 1.14–1.74) for the second (1983–1998) period. The absolute numbers were 0.56, 0.48 and 0.34 per thousand births for the first, second and the last period, respectively.

### 3.1 Baseline Characteristics

Mothers who died within 1 year were younger at first and last childbirth, had lower educational level, and more often had only one child compared to those who survived. Like the surviving mothers, the majority of those who died were born in Norway. However, among those who died, 19.4% had an unknown country of birth (Table S1).

### 3.2 Pregnancy Complications and Associated Maternal Mortality

Pregnancy complications were associated with an increased risk of death within 1 year after childbirth (Table 1). We found higher odds of death if any complication occurred in the last pregnancy compared to any complication in the first. Crude OR for PAM was 4.24, (95% CI 3.53–5.10) for complications occurring in the last pregnancy, whereas 2.52 (2.08–3.06) for complications occurring in the first pregnancy, compared to mothers without complications in any pregnancy. Adjusted estimates are reported for analyses of complications in the last pregnancy. For analyses of complications in the first and in any pregnancy, adjustment for covariates did not substantially change ORs. Thus, unadjusted estimates are presented.

**TABLE 1:** Odds Ratios (ORs) with 95% confidence intervals (CIs) of maternal mortality within 1 year after childbirth in 1 237 254 mothers by pregnancy complications up to eight pregnancies. Medical Birth Registry of Norway, 1967–2019.

Pregnancy complications	Complications in the first pregnancy		Complications in the last pregnancy			Complications in any pregnancy	
	*n*/*N* (%)	Crude OR (95% CI)	*n*/*N* (%)	Crude OR (95% CI)	Adjusted OR (95% CI)	*n*/*N* (%)	Crude OR (95% CI)
None	286/955 076 (0.03)	1 (Reference)	286/955076 (0.03)	1 (Reference)	1 (Reference)	286/955076 (0.03)	1 (Reference)
Pregnancy affected by any complication	174/238190 (0.07)	2.52 (2.08–3.06)	188/148013 (0.13)	4.24 (3.53–5.10)	4.13 (3.40–5.02)	225/282178 (0.08)	2.66 (2.23–3.17)
Placental abruption	8/6110 (0.13)	2.93 (0.73–11.80)	13/5839 (0.22)	3.69 (1.18–11.54)	3.75 (1.20–11.72)	17/13242 (0.13)	3.08 (1.15–8.29)
Preeclampsia *Preterm Term*	44/52725 (0.08) 14/10250 (0.14) 27/40288 (0.07)	3.16 (0.78–12.72) 4.56 (2.66–7.81) 1.95 (1.25–3.03)	43/33516 (0.13) 20/7386 (0.27) 23/26130 (0.09)	2.94 (1.92–4.49) 9.06 (5.75–14.27) 2.25 (1.36–3.73)	4.42 (3.17–6.15) 10.16 (6.42–16.06) 2.88 (1.84–4.50)	57/72251 (0.08) 24/14665 (0.16) 31/57147 (0.05)	3.21 (0.80–12.93) 5.47 (3.60–8.30) 1.56 (0.98–2.48)
Preterm birth	71/76081 (0.09)	2.26 (1.59–3.19)	110/62561 (0.18)	4.45 (3.38–5.84)	4.32 (3.25–5.75)	134/128532 (0.10)	2.77 (2.13–3.61)
Perinatal death	37/14796 (0.25)	8.37 (5.64–12.43)	46/5480 (0.84)	30.07 (22.32–42.41)	24.18 (16.66–35.08)	59/28568 (0.21)	7.63 (5.55–10.49)
SGA < 2.5	36 /44005 (0.08)	2.63 (1.83–3.80)	29/26103 (0.11)	3.16 (2.03–4.92)	2.90 (1.85–4.54)	44/63591 (0.07)	2.37 (1.70–3.30)
GDM	6/12093 (0.05)	1.45 (0.60–3.52)	12/20070 (0.06)	1.64 (0.84–3.17)	1.43 (0.63–3.25)	13/25538 (0.05)	1.42 (0.75–2.67)
Gestational hypertension	10/23416 (0.04)	1.43 (0.76–2.68)	12/19707 (0.06)	2.03 (1.14–3.62)	2.05 (1.12–3.74)	16/39420 (0.04)	1.13 (0.65–1.98)

*Note:* Adjusted for maternal age at first childbirth, education, period of last childbirth (1967–1982, 1983–1998 and 1999–2019), and pre‐pregnancy chronic medical conditions (asthma, rheumatoid arthritis, epilepsy, renal disease, hypertension and diabetes). Reference Category: Mothers Who Had no Complication in any Pregnancy (Up to Eight Pregnancies).

Abbreviations: GDM, gestational diabetes mellitus; *n*, number of deaths; *N*, total; SGA < 2.5, small for gestational age below the 2.5 percentile.

Adjusted OR for maternal mortality was 4.13 (95% CI: 3.40–5.02) for mothers with complications occurring in the last pregnancy compared to mothers without complications. We found associations with maternal mortality for most of the complications when assessed individually. The strongest estimates were found for perinatal death (OR: 24.18, 95% CI: 16.66–35.08), preterm preeclampsia (OR: 10.16, 95% CI: 6.42–16.06), preterm birth (OR: 4.32, 95% CI: 3.25–5.75) and placental abruption (OR: 3.75, 95% CI: 1.20–11.72).

A sensitivity analysis that excluded mothers with late spontaneous abortions (gestational age 16–21 completed weeks) gave similar results as main analyses except for perinatal mortality (Table S2).

### 3.3 Maternal Mortality by Number of Complicated Pregnancies, Stratified by Number of Lifetime Pregnancies (One to Four Pregnancies)

The risk of death within 1 year after pregnancy was dependent on having one or more complicated pregnancies relative no complications across total number of lifetime pregnancies (Table 2). We found higher odds of death in mothers who had one or more complicated pregnancies compared to mothers with two pregnancies and no complications (common reference group). Mothers with one lifetime pregnancy had particularly high risk of death within 1 year after childbirth if this was a complicated pregnancy. Adjusted ORs were 3.88 (95% CI: 2.91–5.18) if no complications, whereas 12.19 (95% CI: 8.81–16.86) if complicated, compared to mothers with two lifetime pregnancies without complications. There was a tendency for mothers with greater numbers of complicated pregnancies to have increased mortality risk (Table 2). For mothers with three and four lifetime pregnancies, the point estimates indicated a possible increasing mortality risk by increasing number of complicated pregnancies. However, low numbers of mothers with more than two complicated pregnancies and the low number of deaths in these groups (three, two and one), resulted in overlapping CIs.

**TABLE 2:** Odds Ratios (ORs) with 95% confidence intervals (CIs) of maternal mortality within 1 year after childbirth in 1 238 056 mothers by number of complicated pregnancies, stratified analyses by number of lifetime pregnancies (one to four pregnancies), Medical Birth Registry of Norway, 1967–2019.

	Mothers with number of lifetime pregnancies (up to four pregnancies)											
	One lifetime pregnancy (*n* = 265)			Two pregnancies (*n* = 144)			Three pregnancies (*n* = 73)			Four pregnancies (*n* = 29)		
Number of complicated pregnancies	*n*/*N* (per 1000)	Crude OR (95% CI)	Adjusted OR (95% CI)*	*n*/*N* (per 1000)	Crude OR (95% CI)	Adjusted OR (95% CI)*	*n*/*N* (per 1000)	Crude OR (95% CI)	Adjusted OR (95% CI)*	*n*/*N* (per 1000)	Crude OR (95% CI)	Adjusted OR (95% CI)*
None	168/237578 (0.71)	4.17 (3.20–5.45)	3.88 (2.91–5.18)	80/471818 (0.17)	1.00 Reference	1.00 Reference	38/213645 (0.18)	1.05 (0.71–1.52)	1.07 (0.72–1.60)	5/41931 (0.12)	0.72 (0.29–1.77)	0.68 (0.28–1.70)
one	97/46763 (2.07)	12.23 (9.10–16.45)	12.19 (8.81–16.86)	52/99567 (0.52)	3.08 (2.17–4.37)	3.20 (2.23–4.59)	24/59814 (0.40)	2.37 (1.50–3.74)	2.25 (1.40–3.60)	17/17550 (0.97)	5.72 (3.39–9.65)	5.23 (3.07–8.92)
Two				12/22310 (0.54)	3.17 (1.73–5.82)	3.04 (1.56–5.89)	8/15039 (0.53)	3.14 (1.52–6.49)	3.10 (1.49–6.45)	4/5600 (0.71)	4.21 (1.54–11.51)	3.69 (1.34–10.14)
Three							3/4036 (0.74)	4.39 (1.38–13.89)	3.10 (0.76–12.64)	2/1839 (1.09)	6.42 (1.58–26.14)	5.87 (1.44–23.98)
Four										1/566 (1.77)	10.44 (1.45–75.13)	10.05 (1.39–72.59)

*Note:* Reference Category: Mothers Who Had Two Pregnancies With no Complications.

Abbreviations: *n*, number of maternal deaths; *N*, total.

* Adjusted for maternal age at first childbirth, education, period of last childbirth (1967–1982, 1983–1998 and 1999–2019), and pre‐pregnancy chronic medical conditions (asthma, rheumatoid arthritis, epilepsy, renal disease, hypertension and diabetes).

### 3.4 Number of Complications in the Last Pregnancy and Maternal Mortality, Stratified by Three Periods of Last Childbirth

Table 3 Shows associations between complications in last pregnancy and PAM across three time periods, restricting analyses to mothers with first pregnancy up to 2013. Overall, risk of maternal death increased by increasing number of complications in the last pregnancy for all three periods. The absolute maternal mortality decreased in the reference group from 0.46 per 1000 births in 1967–1982 to 0.23 per 1000 births in 1999–2019. When using one common reference category across all study periods (mothers with no complications and giving birth in the last period), we found no convincing and consistent trend of mortality. Although relative estimates for the risk associated with one complication was 4.6‐fold in first, 7‐fold in second and 4‐fold in the last period, the respective absolute numbers were 0.94, 1.42 and 0.94 per thousand births. In all periods, the adjusted ORs for mortality increased by increasing number of complications in last pregnancy from none to two. For mothers with three complications in the last pregnancy, the risk increased even more in the first period, whereas not in the other two periods. An important observation was that there were few mothers with three complications who died in the last two periods.

**TABLE 3:** Odds Ratios (ORs) with 95% confidence intervals (CIs) of maternal mortality within 1 year after childbirth in 1 094 242 mothers (with first childbirth included up to 2013 and 485 maternal deaths within 1 year) by number of complications in the last pregnancy, stratified by three periods of last childbirth, Medical Birth Registry of Norway, 1967–2019.

	1967–1982			1983–1998			1999–2019		
Complications in the last pregnancy	*n*/*N* (per 1000)	Crude OR (95% CI)^a^	Crude OR (95% CI)^b^	*n*/*N* (per 1000)	Crude OR (95% CI)^a^	Crude OR (95% CI)^b^	*n*/*N* (per 1000)	Crude OR (95% CI)^a^	Adjusted OR (95% CI)^c^
None	104/225397 (0.46)	1.00 Reference	2.28 (1.74–2.99)	111/323412 (0.34)	1.00 Reference	1.70 (1.30–2.21)	95/415316 (0.23)	1.00 Reference	1.00 Reference
One	23/24535 (0.94)	2.03 (1.29–3.19)	4.63 (2.95–7.27)	49/34591 (1.42)	4.13 (2.95–5.78)	7.00 (4.99–9.82)	46/49064 (0.94)	4.18 (2.88–6.07)	4.14 (2.79–6.13)
Two	7/2837 (2.47)	5.36 (2.49–11.53)	12.21 (5.68–26.26)	16/5115 (3.13)	9.14 (5.41–15.48)	15.49 (9.15–26.22)	19/7627 (2.49)	11.35 (6.62–18.89)	11.50 (6.81–19.43)
Three	9/651 (13.82)	30.37 (15.30–60.27)	69.21 (34.9–137.26)	2/964 (2.07)	6.06 (1.49–24.55)	10.26 (2.53–41.63)	4/1076 (3.72)	13.61 (4.30–43‐14)	9.38 (2.29–38.41)

*Note:* Models: There are three statistical models. In model (a), we used period specific reference category (mothers without complications, 1967–1982; 1983–1998, and 1999–2019) and present crude estimates. In model (b), we used a common reference (mothers without complications, 1999–2019). In the third model (c) we present adjusted estimates (maternal age at first childbirth, education, period of last childbirth, and chronic medical conditions), specifically for the years 1999–2019.

Abbreviations: *n*, number of deaths; *N*, total.

## 4 Discussion

### 4.1 Main Findings

Maternal mortality within 1 year of childbirth decreased across the study period for mothers with first singleton births in Norway between 1967 and 2013. Mothers with perinatal death, preterm preeclampsia, preterm birth or placental abruption had particularly increased risk of maternal death compared to mothers with no complications in any pregnancy, and associations were strongest when complications occurred in the last pregnancy. Having more than one complication in the last pregnancy seemed to be associated with a higher risk of death relative to having none or only one complication. When grouping mothers by total number of lifetime pregnancies, the risk of maternal death increased by increasing number of complicated pregnancies. The risk of maternal death was increased twelve times for mothers with one lifetime pregnancy with complications, relative to mothers with two lifetime pregnancies without complications.

### 4.2 Strengths and Limitations

Strengths of this study include its population‐based design and large sample size covering 53 years of obstetric practice, enabling us to study maternal mortality, a rare outcome. Births to each mother were identified and pregnancy complications were recorded prospectively, reducing the likelihood of selection and recall bias. Any possible misclassification in exposure would tend to be non‐differential and unrelated to the outcome due to the prospective registration. Examining rare pregnancy complications, like placental abruption and perinatal death, also depended on the availability of a large dataset. Additionally, the existing literature has typically focused on the association between pregnancy complications and maternal mortality risk, without considering how this risk might differ based on birth order or parity throughout a woman's entire reproductive history. A complete picture of women's reproductive experience is the unique aspect of our study and the strong association between complications in the last pregnancy and maternal mortality risk is a novel finding.

Information on some potential confounders was lacking, such as smoking, body mass index and clinical characteristics. These risk factors were not registered in the MBRN across the entire time frame of the study. Changes in the reporting format in the MBRN are another limitation. Unlike checkboxes, notification based on free text before 1999 may be linked to underreporting, especially for less severe complications [9]. However, the validity of preeclampsia, one of the complications we studied, has indicated a positive predictive value of 88.3% (births 1967–2002) [16]. Moreover, lack of ethnic diversity may, to some extent, limit the generalisability of our findings to different populations. However, representativeness may not necessarily be a critical factor for scientific studies [25]. The possibility of survivorship bias cannot be ignored. Certain autoimmune conditions such as systemic lupus erythematosus, myotonic dystrophy or venous thromboembolism, may predispose women for certain pregnancy complications through common molecular pathway or genetic risk and are also related to higher risk of PAM [26, 27]. However, these conditions are rare and most likely underreported in the MBRN. Unmeasured confounding remains possible due to the limited information on clinical data and pre‐gestational conditions. We also lacked details about lifestyle and socio‐economic factors, beyond education. Future research should consider more specific clinical and lifestyle variables. For rare and severe outcomes like maternal mortality, audit data are a valuable and necessary supplement [28]. In the UK, a system of confidential enquiries/audits has been implemented as a routine since 1952, to evaluate severe pregnancy outcomes including maternal deaths [29], highlighted as gold standard by the WHO. In the Nordic countries, audits have been done on a research basis for mothers dying within 42 days after delivery [30], and the combination of large registry‐based and detailed audit‐based studies could yield important additional knowledge into this field.

### 4.3 Interpretation

Our findings suggest that mothers with multiple pregnancy complications should be closely monitored. Moreover, optimising postpartum care for such mothers should be a public health priority. Our results could help healthcare providers to target high‐risk mothers for postpartum follow‐up with interventions to reduce risk of death.

Norway has one of the world's lowest maternal mortality rates, although inconsistencies in definitions and underreporting complicate time period comparisons [1]. Overall, there was a decreasing trend of mortality linked to pregnancy complications across the study period. High mortality risk was found in young mothers and in women with low education. Our findings correspond with previous evidence [31, 32], indicating that early childbearing and low education are linked to a range of negative health outcomes.

We found strong associations between maternal death and pregnancy complications in mothers whose last pregnancy was affected by perinatal death, preterm birth, and placental abruption compared to those without complications in any pregnancy. These findings are consistent with results from a Danish population‐based study that suggested an association between perinatal loss and overall maternal mortality within 1 year [33]. Moreover, a Swedish cohort study found that preterm birth was an independent risk factor for maternal mortality [34], and Tikkanen et al. showed that placental abruption was an important cause of maternal death [35]. These investigators [33, 34, 35] concluded that pregnancy complications (such as perinatal death, preterm birth, and placental abruption) are linked to maternal death within 1 year. However, because they did not account for complications across lifetime number of pregnancies, they did not identify complications in mothers' last pregnancy as strongly associated with the risk of death. This is the distinctive feature of our study.

Another finding from our study is that risk of dying was, to some extent, modified by the number of complicated pregnancies. A recent study [36] found that the risk of long‐term cardiovascular mortality increased with an increasing number of births complicated with preeclampsia, placental abruption, low birth weight, stillbirth or preterm birth. This study, however, did not focus on maternal death within 1 year after childbirth.

A dose–response trend was observed with increasing risk of PAM by increasing number of complications in the last pregnancy from none to two. The association between number of complications in the last pregnancy and mortality was stronger in the last period than in the first period. One explanation may be that the birth notification form in the MBRN was changed in 1999 and check boxes were added to free text [9]. This has led to the increased reporting of complications in the last period, which were not as consistently reported in the first period, for example preeclampsia, as previously shown [9]. This means that the reference (denominator) in the first time periods may include mothers with complications (misclassified as uncomplicated), whereas in the last period, mothers in the reference group are less likely to have complications. This is also supported by a much lower absolute mortality rate for the reference group in the last period, compared to the first and the second period. The relative risk estimates were largely unchanged after adjustment for mothers' chronic medical conditions.

## 5 Conclusions

Maternal mortality risk within 1 year after childbirth has decreased in Norway across more than 50 years of obstetric practice. However, both the number and type of pregnancy complications, as well as parity, remain important factors. Complications in the last pregnancy were more strongly associated with PAM than those in the first pregnancy. Although the results were adjusted for potential confounders, residual confounding remains possible. This study shows that events across mothers' complete pregnancy history may better predict maternal mortality risk than relying merely on information from any one pregnancy. Further research is needed to understand the timing and causes of deaths during the extended postpartum period.

## Author Contributions

N.A. and R.S. designed this study. R.S. acquired funding, obtained access to the data, and is a guarantor for data quality. N.A. analysed the data, interpreted the results, wrote the manuscript, and is responsible for the reviewing and proof‐reading of the manuscript. All authors provided valued input to analyses and to the manuscript. All authors have read and approved the final version.

## Conflicts of Interest

The authors declare no conflicts of interest.

## Supporting information


**Table S1.** Baseline characteristics of 1 237 254 mothers with first singleton births between 1967–2019, Medical Birth Registry of Norway.
**Table S2**. Odds Ratios (ORs) with 95% confidence intervals (CIs) of maternal mortality within 1 year after childbirth in 1 235 011 mothers (of 505 maternal deaths within 1 year) by pregnancy complications, restricted to gestational age above 22 weeks, Medical Birth Registry of Norway, 1967–2019.

## Data Availability

The data that support the findings of this study are available on request from the corresponding author. The data are not publicly available due to privacy or ethical restrictions.

## References

[1] S. Vangen , L. Ellingsen , A. B. Andersgaard , et al., “Maternal Deaths in Norway 2005–2009,” Tidsskrift for den Norske Lægeforening 134, no. 8 (2014): 836–839. 10.4045/tidsskr.13.0203 24780982

[2] A. Esscher , U. Högberg , B. Haglund , and B. Essën , “Maternal Mortality in Sweden 1988–2007: More Deaths Than Officially Reported,” Acta Obstetricia et Gynecologica Scandinavica 92, no. 1 (2013): 40–46. 23157437 10.1111/aogs.12037 PMC3565446

[3] A. B. Andersgaard , J. Langhoff‐Roos , and P. Øian , “Direct Maternal Deaths in Norway 1976–1995,” Acta Obstetricia et Gynecologica Scandinavica 87, no. 8 (2008): 856–861. 18607819 10.1080/00016340802237067

[4] WHO, UNICEF, UNNFPA and The World Bank and the United Nations Population Division , Trends in Maternal Mortality 2000–2017 (Geneva: World Health Organization, 2019).

[5] Knoema , “World Data Atlas. Norway–Maternal Mortality Ratio, 2021”.

[6] World Health Organization , “The Global Health Observatory: Maternal Deaths,” https://www.who.int/data/gho/indicator‐metadata‐registry/imr‐details/4622.

[7] R. Tikkanen , Z. Munira , M. Z. Gunja , M. FitzGerald , and L. Zephyrin , Maternal Mortality and Maternity Care in the United States Compared to 10 Other Developed Countries. The Commonwealth Fund, United States 2020, 10.26099/411v-92552020.

[8] R. Skjaerven , A. J. Wilcox , K. Klungsøyr , et al., “Cardiovascular Mortality After Pre‐Eclampsia in One Child Mothers: Prospective, Population Based Cohort Study,” BMJ 27, no. 345 (2012): e7677. 10.1136/bmj.e7677 PMC3508198 23186909

[9] K. Klungsøyr , N. H. Morken , L. Irgens , S. E. Vollset , and R. Skjaerven , “Secular Trends in the Epidemiology of Pre‐Eclampsia Throughout 40 Years in Norway: Prevalence, Risk Factors and Perinatal Survival,” Paediatric and Perinatal Epidemiology 26, no. 3 (2012): 190–198. 22471678 10.1111/j.1365-3016.2012.01260.x

[10] S. Cnattingius , A. Ericson , J. Gunnarskog , and B. Källén , “A Quality Study of a Medical Birth Registry,” Scandinavian Journal of Social Medicine 18, no. 2 (1990): 143–148. 2367825 10.1177/140349489001800209

[11] L. M. Irgens , “The Medical Birth Registry of Norway. Epidemiological Research and Surveillance Throughout 30 Years,” Acta Obstetricia et Gynecologica Scandinavica 79, no. 6 (2000): 435–439. 10857866

[12] L. DeRoo , R. Skjaerven , A. Wilcox , et al., “Placental Abruption and Long‐Term Maternal Cardiovascular Disease Mortality: A Population‐Based Registry Study in Norway and Sweden,” European Journal of Epidemiology 31, no. 5 (2016): 501–511. 26177801 10.1007/s10654-015-0067-9 PMC4901083

[13] F. N. Moth , T. R. Sebastian , J. Horn , J. Rich‐Edwards , P. R. Romundstad , and B. O. Åsvold , “Validity of a Selection of Pregnancy Complications in the Medical Birth Registry of Norway,” Acta Obstetricia et Gynecologica Scandinavica 95, no. 5 (2016): 519–527. 26867143 10.1111/aogs.12868

[14] K. Klungsøyr , Q. E. Harmon , L. B. Skard , et al., “Validity of Pre‐Eclampsia Registration in the Medical Birth Registry of Norway for Women Participating in the Norwegian Mother and Child Cohort Study, 1999–2010,” Paediatric and Perinatal Epidemiology 28, no. 5 (2014): 362–371. 25040774 10.1111/ppe.12138 PMC4167249

[15] L. M. Irgens , “Medical Birth Registry—An Essential Resource in Perinatal Medical Research,” Tidsskrift for den Norske Lægeforening 122, no. 26 (2002): 2546–2549. 12522882

[16] L. C. Thomsen , K. Klungsøyr , L. T. Roten , et al., “Validity of the Diagnosis of Pre‐Eclampsia in the Medical Birth Registry of Norway,” Acta Obstetricia et Gynecologica Scandinavica 92, no. 8 (2013): 943–950. 23621424 10.1111/aogs.12159

[17] Norwegian Institute of Public Health and Research Report 2020 , “The Medical Birth Registry of Norway,” November 2020 version 1.2, https://hdl.handle.net/11250/2723861.

[18] World Health Organization , “Women's Children's and Adolescent's Health: Module on Stillbirth,” 2023, https://pmnch.who.int/resources/tools‐and‐toolkits/stillbirths‐toolkit/kenya/what‐is‐stillbirth.

[19] G. E. Chalouhi , J. P. Bernard , G. Benoist , B. Nasr , Y. Ville , and L. J. Salomon , “A Comparison of First Trimester Measurements for Prediction of Delivery Date,” Journal of Maternal‐Fetal and Neonatal Medicine 24, no. 1 (2011): 51–57. 20350241 10.3109/14767051003728229

[20] R. Skjaerven , H. K. Gjessing , and L. S. Bakketeig , “Birthweight by Gestational Age in Norway,” Acta Obstetricia et Gynecologica Scandinavica 79, no. 6 (2000): 440–449. 10857867

[21] A. J. Wilcox , M. Cortese , D. R. McConnaughey , D. Moster , and O. Basso , “The Limits of Small‐for‐Gestational‐Age as a High‐Risk Category,” European Journal of Epidemiology 36, no. 10 (2021): 985–991. 34661814 10.1007/s10654-021-00810-z PMC12380009

[22] L. J. Kjerpeseth , V. Hjellvik , H. L. Gulseth , et al., “Prevalence and Treatment of Gestational Diabetes in Norway 2010–2020,” Diabetes Research and Clinical Practice 207 (2024): 111025. 38000666 10.1016/j.diabres.2023.111025

[23] Statistics Norway , 2024 https://www.ssb.no/utdanning/utdanningsniva/artikler/fakta‐om‐utdanning.

[24] L.‐A. McNutt , C. Wu , X. Xue , and J. P. Hafner , “Estimating the Relative Risk in Cohort Studies and Clinical Trials of Common Outcomes,” American Journal of Epidemiology 157, no. 10 (2003): 940–943. 12746247 10.1093/aje/kwg074

[25] K. J. Rothman , J. E. Gallacher , and E. E. Hatch , “Why Representativeness Should Be Avoided,” International Journal of Epidemiology 42, no. 4 (2013): 1012–1014. 24062287 10.1093/ije/dys223 PMC3888189

[26] W. M. Merz , R. Fischer‐Betz , K. Hellwig , G. Lamprecht , and U. Gembruch , “Pregnancy and Autoimmune Disease,” Deutsches Ärzteblatt International 119, no. 9 (2022): 145–156. 34874264 10.3238/arztebl.m2021.0353 PMC9201458

[27] F. Aranda , S. Udry , S. Perés Wingeyer , et al., “Maternal Carriers of the ANXA5 M2 Haplotype Are Exposed to a Greater Risk for Placenta‐Mediated Pregnancy Complications,” Journal of Assisted Reproduction and Genetics 35, no. 5 (2018): 921–928. 29497952 10.1007/s10815-018-1142-4 PMC5984886

[28] M. B. K. Knight , D. Tuffnell , R. Patel , et al., Saving Lives, Improving Mothers' Care–Lessons Learned to Inform Maternity Care From the UK and Ireland Confidential Enquiries Into Maternal Deaths and Morbidity 2017–19 (Oxford: National Perinatal Epidemiology Unit, University of Oxford, 2021).

[29] A. M. Weindling , “The Confidential Enquiry Into Maternal and Child Health (CEMACH),” Archives of Disease in Childhood 88, no. 12 (2003): 1034–1037. 14670760 10.1136/adc.88.12.1034 PMC1719387

[30] S. Vangen , B. Bødker , L. Ellingsen , et al., “Maternal Deaths in the Nordic Countries,” Acta Obstetricia et Gynecologica Scandinavica 96, no. 9 (2017): 1112–1119. 28542709 10.1111/aogs.13172

[31] G. L. Jones , C. A. Mitchell , J. E. Hirst , and D. O. C. Anumba , “On Behalf of the Royal College of Obstetricians Gynaecologists. Understanding the Relationship Between Social Determinants of Health and Maternal Mortality,” BJOG: An International Journal of Obstetrics & Gynaecology 129, no. 7 (2022): 1211–1228. 35139580 10.1111/1471-0528.17044

[32] F. Halland , N. H. Morken , L. A. DeRoo , K. Klungsøyr , A. J. Wilcox , and R. Skjærven , “Long‐Term Mortality in Mothers With Perinatal Losses and Risk Modification by Surviving Children and Attained Education: A Population‐Based Cohort Study,” BMJ Open 6, no. 11 (2016): e012894. 10.1136/bmjopen-2016-012894 PMC5168516 27884847

[33] D. Hvidtjørn , C. Wu , D. Schendel , E. Thorlund Parner , and H. T. Brink , “Mortality in Mothers After Perinatal Loss: A Population‐Based Follow‐Up Study,” BJOG: An International Journal of Obstetrics and Gynaecology 123, no. 3 (2016): 393–398. 25565567 10.1111/1471-0528.13268

[34] C. Crump , J. Sundquist , and K. Sundquist , “Preterm Delivery and Long Term Mortality in Women: National Cohort and Co‐Sibling Study,” BMJ 370 (2020): m2533. 32816755 10.1136/bmj.m2533 PMC7436341

[35] M. Tikkanen , M. Gissler , M. Metsäranta , et al., “Maternal Deaths in Finland: Focus on Placental Abruption,” Acta Obstetricia et Gynecologica Scandinavica 88, no. 10 (2009): 1124–1127. 19707898 10.1080/00016340903214940

[36] L. G. Kvalvik , R. Skjærven , G. Sulo , A. Singh , Q. E. Harmon , and A. J. Wilcox , “Pregnancy History at 40 Years of Age as a Marker of Cardiovascular Risk,” Journal of the American Heart Association 13, no. 5 (2024): e030560. 38410997 10.1161/JAHA.123.030560 PMC10944058

