# Supplementary material for: Pregnancy‐Associated Maternal Mortality Within One Year After Childbirth: Population‐Based Cohort Study
Source: BJOG. 2024 Oct 23;132(3):365–74. doi: 10.1111/1471-0528.17985 (PMC11704022; doi:10.1111/1471-0528.17985)
Supplement: Supplementary file 1 — Table S1. Baseline characteristics of 1 237 254 mothers with first singleton births between 1967–2019, Medical Birth Registry of Norway. Table S2. Odds Ratios (ORs) with 95% confidence intervals (CIs) of maternal mortality within 1 year after childbirth in 1 235 011 mothers (of 505 maternal deaths within 1 year) by pregnancy complications, restricted to gestational age above 22 weeks, Medical Birth Registry of Norway, 1967–2019. [file BJO-132-365-s001.docx]

**Table S1:** Baseline characteristics of 1 237 254 mothers with first singleton births between 1967 to 2019, Medical Birth Registry of Norway.

| **Characteristics** | **Died within one year after childbirth.**  **(n= 511)** | **Alive one year after childbirth.**  **(N=1 236 743)** |
| --- | --- | --- |
| Mothers’ age at first childbirth (years)  *≤ 20*  *21-24*  *25-29*  *30-34*  *35-39*  *≥ 40* | 80 (15.66)  188 (36.79)  139 (27.20) 65 (12.72)  35 (6.85) 4 (0.78) | 133 868 (10.82)  433 648 (35.06)  411 289 (33.26)  192 351 (15.55)  55 723 (4.51)  9 864 (0.80) |
| Mothers’ age at last childbirth (years)  *≤ 20*  *21-24*  *25-29*  *30-34*  *35-39*  *≥ 40* | 25 (4.89)  117 (22.90)  157 (30.72)  110 (21.53)  78 (15.26)  24 (4.70) | 15 044 (1.22)  136 692 (11.05)  365 162 (29.53)  437 307 (35.36)  231 181 (18.69)  51 357 (4.15) |
| Educational level (years)  *< 11*  *11-13*  *≥14*  ^*^Missing | 187 (36.59)  181 (35.42)  94 (18.40)  49 (9.59) | 231 144 (18.69)  480 021 (38.81)  505 866 (40.90)  19 712 (1.59) |
| Mothers’ country of birth  *Norway*  *International*  ^*^Missing | 362 (70.84)  50 (9.78)  99 (19.37) | 1 084 884 (87.72)  123 981 (10.02)  27 878 (2.25) |
| Mother with number of births  *One lifetime*  *Two*  *Three*  *Four and more* | 258 (50.49)  144 (28.18)  73 (14.29)  36 (7.05) | 265 644 (21.48)  593 551 (47.99)  292 461 (23.65)  85 087 (6.88) |

Numbers are expressed in frequency (percentage).

* Out of 27 977 mothers with missing information on country of birth, 9.91% (n=2773) had missing information on education.

**Table S2:** Odds Ratios (ORs) with 95% confidence intervals (CIs) of maternal mortality within one year after childbirth in 1 235 011 mothers (of 505 maternal deaths within one year) by pregnancy complications, restricted to gestational age above 22 weeks, Medical Birth Registry of Norway, 1967 to 2019.

| **Pregnancy**  **complications** | **Complications in the first pregnancy** | | **Complications in the last pregnancy** | | | **Complications in any pregnancy** | |
| --- | --- | --- | --- | --- | --- | --- | --- |
|  | **n/N**  **(%)** | **Crude OR**  **(95% CI)** | **n/N**  **(%)** | **Crude OR**  **(95% CI)** | **Adjusted OR**  **(95% CI)** | **n/N**  **(%)** | **Crude OR**  **(95% CI)** |
| None | 264/ 912 721  (0.03) | 1 (Reference) | 286/955 076  (0.03) | 1 (Reference) | 1 (Reference) | 286/955 076  (0.03) | 1 (Reference) |
| Pregnancy affected by any complication | 169/237 335  (0.07) | 2.46  (2.03- 2.98) | 182/145 770  (0.12) | 4.17  (3.46- 5.02) | 4.05  (3.33- 4.94) | 219/279 935  (0.08) | 2.61  (2.19- 3.11) |
| Placental abruption | 8/6075  (0.13) | 2.94  (0.73- 11.84) | 13/5787  (0.22) | 3.69  (1.18- 11.54) | 3.74  (1.19- 11.70) | 17/13 150  (0.13) | 3.09  (1.15-8.29) |
| Preeclampsia  *Preterm*  *Term* | 44/52 637  (0.08)  14/10 233  (0.14)  27/40 222  (0.07) | 3.17  (0.79- 12.76)  4.57  (2.67- 7.82)  1.95  (1.25- 3.04) | 43/33 507  (0.13)  20/7377  (0.27)  23/26 130  (0.09) | 2.94  (1.92-4.49)  9.07  (5.76- 14.28)  2.94  (1.92- 4.49) | 4.40  (3.16- 6.13)  10.09  (6.38- 15.97)  2.87  (1.83- 4.48) | 57/72 139  (0.08)  24/14 637  (0.16)  31/57 062  (0.05) | 3.21  (0.80-12.93)  5.48  (3.61-8.31)  1.56  (0.98-2.48) |
| Preterm birth | 66/75 535  (0.09) | 2.27  (1.60- 3.20) | 104/60 318  (0.17) | 4.52  (3.44- 5.94) | 4.39  (3.30- 5.84) | 128/126 289  (0.10) | 2.80  (2.15-3.64) |
| Perinatal death | 32/14 408  (0.22) | 7.37  (4.82- 11.28) | 40/4067  (0.98) | 38.12  (26.40- 55.04) | 29.26  (19.67-43.51) | 53/27 118  (0.20) | 7.22  (5.16-10.09) |
| SGA <2.5 | 35/43 909  (0.08) | 2.56  (1.76- 3.71) | 28/26 017  (0.11) | 3.02  (1.92-4.75) | 2.76  (1.74- 4.37) | 43/63 387  (0.07) | 2.32  (1.66-3.24) |
| GDM | 6/12 062  (0.05) | 1.46  (0.60- 3.53) | 12/20 056  (0.06) | 1.63  (0.84- 3.17) | 1.41  (0.62- 3.20) | 13/25 487  (0.05) | 1.42  (0.75-2.67) |
| Gestational hypertension | 10/23 381  (0.04) | 1.43  (0.76- 2.68) | 12/19 696  (0.06) | 2.03  (1.14- 3.63) | 2.04  (1.11- 3.74) | 16/39 358  (0.04) | 1.13  (0.65-1.98) |

Abbreviations: SGA <2.5, small for gestational age below the 2.5 percentile; GDM, gestational diabetes mellitus; n, number of deaths; N, Total.

Adjusted for maternal age at first childbirth, education, period of last childbirth (1967-82; 1983-98; 1999-2019), and pre-pregnancy chronic medical conditions (asthma, rheumatoid arthritis, epilepsy, renal disease, hypertension, and diabetes).

Reference category: Mothers who had no complication in any pregnancy (up to eight pregnancies).
